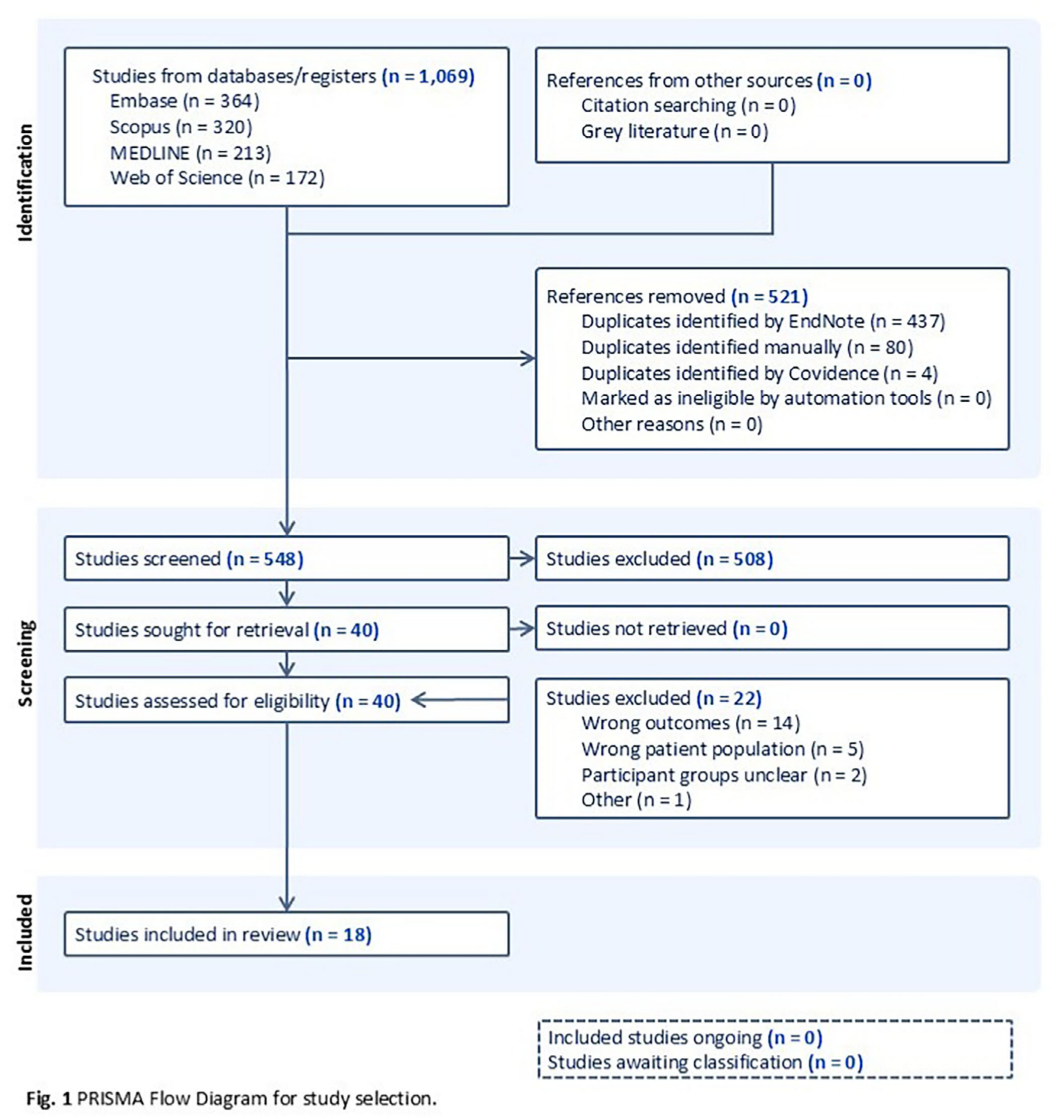# Biomechanical assessments of functional activities in Alzheimer's Disease and Related Dementia: A Scoping Review

**DOI:** 10.1002/alz70856_101805

**Published:** 2025-12-25

**Authors:** Ben Senderling, Amy Kwok, Annalisa Na

**Affiliations:** ^1^ Drexel University, Philadelphia, PA, USA

## Abstract

**Background:**

Assessing joint biomechanics during functional activities can guide interventions by describing altered forces within the musculoskeletal system and affected movement. Growing research indicates functional activities, commonly gait, are potential ADRD biomarkers. However, such studies primarily focused on spatiotemporal parameters, including stride length and cadence. Biomechanics drive spatiotemporal parameters, potentially providing insights to guide treatment. Though the extent biomechanics of such activities are examined and reported in the literature is unclear. This scoping review aims to summarize the biomechanics of functional activities in older adults with ADRD.

**Method:**

We conducted a scoping review as described by Arskey and O’Malley (2005) and recommended by the Preferred Reporting Items for Scoping reviews and Meta‐Analyses Adaptation (PRISMA‐ScR). Online databases (Ovid, Embase, Scopus and Web of Science) were searched from inception to 12/12/2024 using relevant keywords. Eligible studies written in English quantified joint biomechanics during functional activities in older adults (age≥65) living with ADRD. Search results were imported into the web application Covidence. Two authors (BA and AK) screened (titles, abstract, full‐text) and charted studies independently, then reconciled discrepancies through discussion.

**Result:**

Our search yielded 1,069 studies, with 18 meeting criteria (Figure 1). All but one study was published after 2016. Most studies used a cross‐sectional design (*n* = 16) with one longitudinal and one randomized control trial. Extracted average participant characteristics included ranges for age (65.7‐82.6 years) and mild cognitive impairment (Montreal Cognitive Assessment=18.5‐26.1). Functional activities included walking (*n* = 13), Sit‐to‐Stand (*n* = 4) and Timed Up‐and‐Go (*n* = 2). Studies assessed biomechanics using motion capture (*n* = 11) or inertial sensors (*n* = 7) to quantify the ankle (angles:10; moments:4), knee (angles:8; moments:4), and hip (angles:3; moments:4).

**Conclusion:**

Most studies were recently published, assessed lower extremity biomechanics during walking in older adults with MCI. It was beyond the scope of this abstract to synthesize findings. However, our findings suggest a systematic evaluation of walking biomechanics among older adults with MCI. A systematic review may provide further insights to understanding walking as an ADRD biomarker and, ultimately inform intervention strategies for ADRD.